# A needle in a haystack? The impact of a targeted epilepsy gene panel in the identification of a treatable but rapidly progressive metabolic epilepsy: CLN2 disease

**DOI:** 10.1055/s-0044-1786854

**Published:** 2024-05-19

**Authors:** Charles Marques Lourenço, Juliana Maria Ferraz Sallum, Alessandra Marques Pereira, Paula Natale Girotto, Fernando Kok, Daniel Reda Fenga Vilela, Erika Barron, André Pessoa, Bibiana Mello de Oliveira

**Affiliations:** 1 Faculdade de Medicina de São José do Rio Preto, São José do Rio Preto SP, Brazil.; 2 Universidade Federal de São Paulo, Escola Paulista de Medicina, Departamento de Oftalmologia e Ciências Visuais, São Paulo SP, Brazil.; 3 Hospital Moinhos de Vento, Porto Alegre RS, Brazil.; 4 Santa Casa de São Paulo, Faculdade de Ciências Médicas, São Paulo SP, Brazil.; 5 Mendelics Análise Genômica, São Paulo SP, Brazil.; 6 BioMarin Brasil Farmacêutica Ltda., São Paulo SP, Brazil.; 7 Hospital Albert Sabin, Fortaleza CE, Brazil.; 8 Universidade Estadual do Ceará, Fortaleza CE, Brazil.; 9 Universidade Federal do Rio Grande do Sul, Porto Alegre RS, Brazil.

**Keywords:** Neuronal Ceroid Lipofuscinoses, Neurodegenerative Diseases, Epilepsy, Diagnosis, Lipofuscinoses Ceroides Neuronais, Doenças Neurodegenerativas, Epilepsia, Diagnóstico

## Abstract

**Background** Neuronal ceroid lipofuscinoses (NCL) are a group of autosomal recessive, inherited, lysosomal, and neurodegenerative diseases that causes progressive dementia, seizures, movement disorders, language delay/regression, progressive visual failure, and early death. Neuronal ceroid lipofuscinosis type 2 (CLN2), caused by biallelic pathogenic variants of the *TPP1* gene, is the only NCL with an approved targeted therapy. The laboratory diagnosis of CLN2 is established through highly specific tests, leading to diagnostic delays and eventually hampering the provision of specific treatment for patients with CLN2. Epilepsy is a common and clinically-identifiable feature among NCLs, and seizure onset is the main driver for families to seek medical care.

**Objective** To evaluate the results of the Latin America Epilepsy and Genetics Program, an epilepsy gene panel, as a comprehensive tool for the investigation of CLN2 among other genetic causes of epilepsy.

**Methods** A total of 1,284 patients with epilepsy without a specific cause who had at least 1 symptom associated with CLN2 were screened for variants in 160 genes associated with epilepsy or metabolic disorders presenting with epilepsy through an epilepsy gene panel.

**Results** Variants of the *TPP1* gene were identified in 25 individuals (1.9%), 21 of them with 2 variants. The 2 most frequently reported variants were p.Arg208* and p.Asp276Val, and 2 novel variants were detected in the present study: p.Leu308Pro and c.89 + 3G > C Intron 2.

**Conclusion** The results suggest that these genetic panels can be very useful tools to confirm or exclude CLN2 diagnosis and, if confirmed, provide disease-specific treatment for the patients.

## Resumo

**Antecedentes** As lipofuscinoses ceroides neuronais ( *neuronal ceroid lipofuscinoses* , NCLs, em inglês) são um grupo de doenças autossômicas recessivas, hereditárias, lisossomais e neurodegenerativas que causam demência progressiva, crises epiléticas, distúrbios de movimento, atraso/regressão da linguagem, deficiência visual progressiva e morte precoce. A lipofuscinose ceroide neuronal tipo 2 ( *neuronal ceroid lipofuscinosis type 2* , CLN2, em inglês), causada por variantes patogênicas bialélicas do gene *TPP1* , é a única com terapia-alvo aprovada. O diagnóstico laboratorial é realizado por testes específicos, o que leva a atrasos diagnósticos e, consequentemente, prejudica a disponibilização de tratamento. A epilepsia é uma característica comum e clinicamente identificável entre as NCLs, e o início das convulsões é o principal motivo para as famílias buscarem atendimento médico.

**Objetivo** Avaliar os resultados do Programa de Epilepsia e Genética da América Latina, um painel genético, como uma ferramenta abrangente para a investigação de CLN2 entre outras causas genéticas de epilepsia.

**Métodos** Um total de 1.284 pacientes com epilepsia sem uma causa específica e que tinham pelo menos 1 sintoma associado à CLN2 foram rastreados em busca de variantes em 160 genes associados à epilepsia ou a distúrbios metabólicos que apresentam epilepsia, por meio de um painel genético.

**Resultados** Variantes do gene *TPP1* foram identificadas em 25 indivíduos (1,9%), sendo que ; 21 apresentavam duas variantes. As duas variantes mais frequentes foram p.Arg208* e p.Asp276Val, e duas variantes novas foram detectadas neste: p.Leu308Pro e c.89 + 3G > C Intron 2.

**Conclusão** Os resultados sugerem que os painéis genéticos de epilepsia podem ser uma ferramenta útil para confirmar ou excluir o diagnóstico de CLN2 e, se confirmado, fornecer tratamento específico para os pacientes.

## INTRODUCTION

Neuronal ceroid lipofuscinoses (NCLs) are a group of rare lysosomal storage disorders, usually presenting autosomal recessive inheritance. As a group, NCLs are the most common type of inherited neurodegenerative disease in childhood, although the age of onset may vary from infancy to adulthood. Infantile forms are clinically characterized by epileptic seizures, loss of speech, vision, cognitive and motor skills, and early death. 1 Currently, 14 NCL types have been identified: *CLN1* , *CLN2* , *CLN3* , *CLN4* , *CLN5* , *CLN6* , *CLN7* , *CLN8* , *CLN9* , *CLN10* , *CLN11* , *CLN12* , *CLN13* , and *CLN14* ; however, to date, neuronal ceroid lipofuscinosis type 2 (CLN2) is the only NCL with an approved targeted therapy. 2

Neuronal ceroid lipofuscinosis type 2 is caused by biallelic pathogenic variants of the *TPP1* gene, leading to a deficiency of the lysosomal tripeptidyl-peptidase 1 (TPP1) enzyme. Children with classic CLN2 disease commonly have their first seizures between the ages of 2 and 4 years, in most cases preceded by delayed language development and followed by rapid progressive neurodegeneration and loss of motor function. 3 4 However, patients diagnosed with CLN2 may have an onset and course of the disease that deviates from this classic clinical presentation. Atypical cases associated with early or late onset of symptoms, variations in the disease course, and multiple clinical presentations, including ataxia-predominant forms (autosomal recessive spinocerebellar ataxia type 7, SCAR7), have been reported, suggesting a wide phenotypic spectrum for this disease. Therefore, in the general context, this is the spectrum of “TPP1 deficiency”, which involves the severe classic forms, the late forms, and SCAR7. 3 5

A comprehensive South-American study 5 analyzed 30 patients with an atypical form of CLN2 disease and found the first symptoms to be seizures and language abnormalities, which usually occurred approximately at the age of 6 years. In addition, the authors 5 found that seizure onset was the main driver for families to seek medical care. In total, 41% of the patients with documented age of the onset of the first symptom experienced at least one additional symptom. Besides, most patients who presented more than 1 symptom within 12 months had seizures as the first symptom.

Therefore, epilepsy is a common and clinically identifiable feature among NCLs. Epilepsy is a neurological disease defined as at least 2 unprovoked (or reflex) seizures, occurring > 24 hours apart, or 1 unprovoked (or reflex) seizure in a person with a probability ≥ 60% of having a second seizure during the next 10 years, or a previous diagnosis of epilepsy syndrome by a qualified medical professional. 6 7 To date, ∼ 880 human genes and more than 50 copy number variations (CNVs) have been associated with epilepsy or seizures, 8 9 10 11 including metabolic genetic diseases, such as the NCLs. 12

The laboratory diagnosis of CLN2 disease is established through the identification of low TPP1 enzyme activity in dried blood spots (DBSs), fibroblasts, or leukocytes, and the identification of biallelic pathogenic variants in the *TPP1* gene. 13 14 15 Considering that these tests are highly specific, the physician must have suspected CLN2 to request the enzyme or genetic analysis. Therefore, due to the non-specificity of the clinical manifestations, the phenotypic heterogeneity, and the high prevalence of atypical CLN2, diagnostic delays are extremely common in Latin America, which eventually hampers the provision of specific treatment for patients. 13 A recently-published Brazilian consensus on the clinical management and diagnosis of CLN2 16 states that the best strategy for the genetic investigation of CLN2 is to perform a gene panel analysis.

A sponsored testing program called Behind the Seizure was performed in the United States, and its results were published in 2022. Children aged from 0 to 60 months at the time of testing with unprovoked seizure onset were eligible to enter the program, which comprised a multigene epilepsy panel of 125 to 183 genes. The authors 17 found that the genetic panel was helpful to increase the diagnostic yield for CLN2 disease and shorten the patients' diagnostic odyssey, enabling earlier intervention.

We herein report the results of the Epilepsy and Genetics Program, created in 2018 to provide a more comprehensive tool for the investigation of CLN2 among other genetic causes of epilepsy in Latin America. In this program, patients from Argentina, Brazil, Chile, and Mexico with epilepsy without a specific cause and who had at least 1 symptom associated with CLN2 were screened for variants in 160 genes associated with epilepsy or metabolic disorders presenting with epilepsy. Unlike the Behind the Seizure program, the Epilepsy and Genetics Program screened patients with two or more symptoms associated with CLN2 (seizures and another symptom).

## METHODS

### Program design and eligibility

The Epilepsy and Genetics Program was sponsored by BioMarin Pharmaceutical Inc. (Novato, CA, United States) in partnership with Mendelics Análise Genômica SA (São Paulo, SP, Brazil). The patients eligible for testing were those who presented with their first epileptic seizure and/or myoclonus between 2 and 4 years of age and who had at least 1 of the following symptoms: delay or involution in language; neuropsychomotor developmental (NPMD) regression; movement disorders (dystonia, myoclonus, spasticity); ataxia; cerebellar atrophy (detected by magnetic resonance imaging, MRI); and electroencephalogram (EEG) abnormalities (photosensitivity to intermittent low-frequency photic stimulation, of 1 Hz to 2 Hz). Patients with epilepsy with known structural etiology or acute symptomatic seizures in isolation or delayed moderate to severe NPMD regression since birth were not eligible for testing under the program. Data from the patients' genetic profile screened from March 2018 to January 2021 were retrospectively obtained from the Mendelics record system. The physicians responsible for evaluating the clinical characteristics of the patients were required to sign a form stating that they met the eligibility criteria.

### Gene panel, sample collection, and next-generation sequencing analysis

The Epilepsy and Genetics Program screened for variants in 160 genes associated with epilepsy or metabolic disorders presenting with epilepsy, including *TPP1* ( Supplementary Material Chart 1 ; online only: https://www.arquivosdeneuropsiquiatria.org/wp-content/uploads/2024/04/ANP-2024.0016-Supplementary-Material.docx ). The evaluation relied on next-generation sequencing (NGS) technology to assist in the diagnosis of the genetic bases of epilepsy in children with the aforementioned clinical profile.

Genomic DNA was extracted from oral mucosa samples collected by the primary care physician during a routine consultation, using swab tubes provided by Mendelics. The patients were asked to perform oral hygiene followed by absolute fasting for 30 minutes prior to sample collection. For collection, the patients were swabbed ten times inside the right and left cheeks. The samples were sent to Mendelics for analysis. For the NGS, a library was containing all coding exons of the 160 genes included in the panel was developed. The panel was run using the NGS platform of Mendelics (Illumina HighSeq 4000 and/or NovaSeq 6000, Thermo Fisher Scientific, Waltham, MA, United States) according to their protocols, and alignment with the human reference genome using the Burrows-Wheeler Aligner with maximal exact matches (BWA-MEM algorithm; open source). Variant genotyping was performed with the Genome Analysis Toolkit (GATK; Broad Institute, Cambridge, MA, United States) according to the Broad Institute's best practices, as well as algorithmic genotyping of CNVs (large insertions and duplications). Validation of specific variants by Sanger sequencing or multiplex ligation-dependent probe amplification (MLPA) was performed as determined by the Mendelics team, if needed. The annotation and variant pathogenicity prediction were performed using the Mendelics' Abracadabra genetic analysis platform as well as the Human Genome Mutation Database (Cardiff University, Cardiff, Wales, United Kingdom) and by checking the ClinVar (freely accessible public archive) variants classification, associated with control population frequencies. Detected variants were classified according to the criteria of the American College of Medical Genetics (ACMG), 18 with Genome Reference Consortium Human Build 38 (hg38) as a reference.

### Statistical analysis

Calculation of allele frequencies, means, and standard deviations was performed using Microsoft Excel (Microsoft Corp., Redmond, WA, United States) spreadsheet.

## RESULTS

### Molecular analysis

Between March 2018 and January 2021, 1,284 individuals were screened (105 in Argentina; 1,003 in Brazil; 172 in Chile; and 4 in Mexico). A total of 578 variants were identified in 87 genes related to epilepsy or metabolic disorders presenting with epilepsy. Variants in the *SCN1A* , *TPP1* , *MECP2* , *SCN2A* , *SLC2A1* , *UBE3A* , *CLN6* , *PLA2G6* , *MFSD8* , and *CLN8* genes were the most commonly observed ( Table 1 ).

**Table 1:** Number of individuals carrying at least one variant per gene included in the Epilepsy and Genetics Program ( *n* = 425)

Gene	Individuals*	Gene	Individuals*	Gene	Individuals*
*SCN1A*	67	*KCNA2*	3	*DNM1*	1
*TPP1*	25	*KCNQ2*	3	*EPM2A*	1
*MECP2*	23	*KCTD7*	3	*FA2H*	1
*SCN2A*	22	*PPT1*	3	*FOXG1*	1
*SLC2A1*	19	*WDR45*	3	*GNAO1*	1
*UBE3A*	18	*WWOX*	3	*GRIN2D*	1
*CLN6*	17	*ADSL*	2	*HCN1*	1
*PLA2G6*	15	*ARSA*	2	*HNRNPU*	1
*MFSD8*	14	*ATM*	2	*KCNC1*	1
*CLN8*	13	*DEPDC5*	2	*KCNJ10*	1
*CACNA1A*	9	*EEF1A2*	2	*KCNMA1*	1
*KCNT1*	9	*FOLR1*	2	*MEF2C*	1
*SCN8A*	9	*GLB1*	2	*MYH7*	1
*CDKL5*	8	*GRIN1*	2	*NEXMIF*	1
*CLN5*	8	*GRIN2B*	2	*NGLY1*	1
*PCDH19*	8	*GRN*	2	*NHLRC1*	1
*STXBP1*	8	*IQSEC2*	2	*PIGN*	1
*ATP1A3*	7	*PRRT2*	2	*POLG*	1
*CLN3*	7	*SATB2*	2	*PURA*	1
*SYNGAP1*	6	*SLC6A8*	2	*ROGDI*	1
*GABRG2*	5	*SPTAN1*	2	*SCARB2*	1
*SLC6A1*	5	*TSC1*	2	*SCN3A*	1
*GABRA1*	4	*TSC2*	2	*SCN9A*	1
*GABRB3*	4	*ALG13*	1	*SLC12A5*	1
*GAMT*	4	*ATP1A2*	1	*SLC25A12*	1
*GRIN2A*	4	*ATRX*	1	*SLC35A2*	1
*KCNB1*	4	*BRAT1*	1	*SMC1A*	1
*ARX*	3	*CASK*	1	*SYN1*	1
*CHD2*	3	*CLCN4*	1	*VARS2*	1

Note: *Number of individuals carrying variants. Some subjects are carriers of more than one variant.

Variants in the *TPP1* gene were identified in 25 (1.9%) individuals, 21 of whom with 2 variants: 16 from Brazil (64%), 4 from Chile (16%), 3 from Argentina (12%), and 2 from Mexico (8%). Of all *TPP1* variants found ( *n* = 46), 71.7% ( *n* = 33) were classified as pathogenic variants, 19.6% ( *n* = 9), as likely-pathogenic variants, and 8.7% ( *n* = 4), as variants of uncertain significance (VUSs) ( Table 2 ); 17 were unique variants (36.9%).

**Table 2:** Demographic characteristics of individuals with at least one *TTP1* variant identified through the Epilepsy and Genetics Program

Cohort characteristics		Individuals ( *n* = 25)
**Country: % (n)**	Brazil	64% (16)
	Chile	16% (4)
	Argentina	12% (3)
	Mexico	8% (2)
**Sex: % (n)**	Female	56% (14)
	Male	44% (11)
**Days from collection to results: mean ± standard deviation**		25.5 ± 12.14
**Zygosity (** ***TPP1*** **): % (n)**	Homozygosis	48% (12)
	Two different *TPP1* variants	36% (9)
	One *TPP1* variant and one wild-type allele	16% (4)
***TPP1*** **variants (46 alleles): % (n)**	Pathogenic	71.7% (33)
	Likely-pathogenic	19.6% (9)
	Variant of uncertain significance	8.7% (4)

In total, 41% ( *n* = 12) of the patients with at least 1 *TPP1* variant were homozygotes (11 presented 2 pathogenic variants, and 1, 2 likely-pathogenic variants), 36% ( *n* = 9) presented 2 different variants (combinations of pathogenic variants, pathogenic and likely-pathogenic variants, or combinations of VUS variants were observed), and 16% ( *n* = 4) presented 1 variant (patients carrying a VUS variant or a pathogenic variant were observed) and 1 wild-type allele ( Tables 2 and 3 ). The allelic frequencies of each variant in this cohort are shown in Table 4 .

**Table 3:** *TPP1* genotypes identified through the Epilepsy and Genetics Program ( *n* = 25)

Subject	Variant 1	Pathogenicity	Variant 2	Pathogenicity
1	p.Arg208*	Pathogenic	Intronic 5bp from 5′SS	Likely-pathogenic
2	p.Arg208*	Pathogenic	p.Arg208*	Pathogenic
3	p.Asp276Val	Pathogenic	Intronic 1bp from 5′SS	Pathogenic
4	p.Asp276Val	Pathogenic	p.Asp276Val	Pathogenic
5	p.Asp276Val	Pathogenic	c.887–10A > G	Likely-pathogenic
6	p.Asp276Val	Pathogenic	p.Asp276Val	Pathogenic
7	p.Glu302Lys	Likely-pathogenic	p.Leu354Pro	Likely-pathogenic
8	p.Ser475Leu	Pathogenic	p.Arg350Trp	Likely-pathogenic
9	c.887–10A > G	Likely-pathogenic	c.887–10A > G	Likely-pathogenic
10	p.Ser475Leu	Pathogenic	p.Arg350Trp	Likely-pathogenic
11	p.Arg206His	Pathogenic	p.Arg206His	Pathogenic
12	p.Arg206His	Pathogenic	p.Arg206His	Pathogenic
13	c.509–1G > C Intron 5	Pathogenic	c.509–1G > C Intron 5	Pathogenic
14	p.Arg208*	Pathogenic	p.Arg208*	Pathogenic
15	p.Arg208*	Pathogenic	p.Arg208*	Pathogenic
16	c.509–1G > C Intron 5	Pathogenic	c.509–1G > C Intron 5	Pathogenic
17	p.Arg208*	Pathogenic	c.509–1G > C Intron 5	Pathogenic
18	p.Asp276Val	Pathogenic	p.Asp276Val	Pathogenic
19	c.887–10A > G	Likely-pathogenic	p.Arg208*	Pathogenic
20	p.Arg208*	Pathogenic	p.Arg208*	Pathogenic
21	p.Leu308Pro	VUS	c.89 + 3G > C Intron 2	VUS
22	p.Arg497Cys	VUS	_	_
23	p.Ser62Gly	Pathogenic	_	_
24	p.Gln306	VUS	_	_
25	p.Gly300Asp	Pathogenic	_	_

Abbreviation: VUS, variant of uncertain significance.

**Table 4:** Frequency of *TPP1* variants found through the Epilepsy and Genetics Program ( *n* = 25 subjects)

Variants	Genomic coordinates ^#^	Number of alleles	Frequency (%)	Pathogenicity
p.Arg208*	Chr11:6617040	11	23.9	Pathogenic
p.Asp276Val	Chr11:6637951	8	17.4	Pathogenic
c.509–1G > C Intron 5	Chr11:6617154	5	10.9	Pathogenic
c.887–10A > G	Chr11:6616513	4	8.7	Likely-pathogenic
p.Arg206His	Chr11:6617045	4	8.7	Pathogenic
p.Arg350Trp	Chr11:6616342	2	4.3	Likely-pathogenic
p.Ser475Leu	Chr11:6615172	2	4.3	Pathogenic
c.89 + 3G > C Intron 2	Chr11:6619193	1	2.2	VUS
Intronic 1bp from 5′SS	Chr11:6638205	1	2.2	Pathogenic
Intronic 5bp from 5′SS	Chr11:6638852	1	2.2	Likely-pathogenic
p.Glu302Lys	Chr11:6637717	1	2.2	Likely-pathogenic
p.Leu308Pro	Chr11:6616467	1	2.2	VUS
p.Leu354Pro	Chr11:6637560	1	2.2	Likely-pathogenic
p.Arg497Cys	Chr11:6636159	1	2.2	VUS
p.Ser62Gly	Chr11:6640050	1	2.2	Pathogenic
p.Gln306	Chr11:6637703	1	2.2	VUS
p.Gly300Asp	Chr11:6616490	1	2.2	Pathogenic

Abbreviation: VUS, variant of uncertain significance.

Note: ^#^ Genome Reference Consortium Human Build 38 (hg38).

Of the 21 individuals with 2 variants, 2 were siblings (from Mexico) and presented the same variants, and 1 had a monozygotic twin (from Brazil). Genetic sequencing was performed in only one of them, since they share almost all their genetic variants 19 and the same symptoms that led to the diagnostic test.

The 2 most frequently reported variants were p.Arg208* and p.Asp276Val, identified in 23.9% and 17.4% of tested subjects respectively. Two novel variants were detected in the present study: p.Leu308Pro and c.89 + 3G > C Intron 2; however, the impact of these variants on *TPP1* structure, activity and phenotype must be further evaluated.

## DISCUSSION

A recently published Brazilian Consensus on clinical management and diagnosis of CLN2 16 states that patients aged 2 to 4 years presenting language delay and epileptic seizures increasing in frequency despite treatment should be tested with an NGS epilepsy panel, even if the EEG presents nonspecific findings and the brain MRI is normal. As the epilepsy panel covers genes associated with various etiologies of epilepsy, including the different forms of NCLs, it may be a very useful tool for the differential diagnosis and to enable the start of disease-specific treatment, when available.

*TPP1* variants were the second most common in this cohort tested through the Epilepsy and Genetics Program, only less frequent than *SCN1A* variants ( Table 1 ). The proportion of *TPP1* variants was lower than the one found by Gall et al. 21 and higher than the one found by Leal-Pardinas et al. 17 These differences observed between the studies are probably related to the genetic background of each population and/or different inclusion criteria.

The most frequent *TPP1* variants in this cohort were p.Arg208* (23.9%) followed by p.Asp276Val (17.4%) and c.509–1G > C Intron 5 (10.9%). Interestingly, p.Arg208* was observed exclusively in the subset of Brazilian patients, while p.Asp276Val was observed in all patients from the Argentinean subset and in some patients from the Brazilian subset. In accordance with our results, a previous study 2 with South American and Caribbean NCL patients found the most frequent variants to be p.Asp276Val, followed by p.Pro295_Gly296insGluAsnPro and p.Arg208*, in a cohort with a high number of Argentinian and Brazilian patients.

In a review of *TPP1* gene variants, Gardner et al. 20 evaluated 389 individuals from different parts of the world (data from the University College London *TPP1* Locus-specific combined with literature searches) and found 131 unique variants. The 2 most frequently reported variants in this global sample were c.509–1 G > C (27%) and c.622 C > T [p.(Arg208*)] (23%), while the allelic frequency of p.Asp276Val was only found in 2%. Thus, when comparing our results in combination with the South American and Caribbean study 2 to the global report, 20 p.Asp276Val seems to be a regional-specific variant of interest for CLN2 diagnosis in Latin America.

The gold-standard laboratory diagnosis recommended to confirm clinical suspicion of CLN2 disease is the identification of causative mutations in each allele of the *TPP1* gene, and demonstration of deficient TPP1 enzyme activity (in leukocytes, fibroblasts, or DBSs). Molecular confirmation of CLN2 disease is determined by the presence of two *TPP1* pathogenic variants *in trans* ; patients with *TPP1* biallelic variants carrying at least one VUS or likely-pathogenic variant should be referred for biochemical analysis to confirm diagnosis. 15

In the present study, 2 *TPP1* variants were found in 21 subjects, 13 of whom (62%) had two pathogenic variants ( Table 3 ), which determined a molecular diagnosis of CLN2. For 8 patients (38%) whose variants were not both pathogenic, it was recommended that the physician should request subsequent TPP1 enzyme testing to confirm CLN2 diagnosis, based on the global guidelines. 15 The main limitations of the present study are the absence of clinical data to investigate associations with age at symptom onset, age at diagnosis, or genotype-phenotype correlations, as well as the lack of follow-up on enzymatic assays.

However, the recently published Brazilian Consensus on CLN2 16 considers that biallelic pathogenic or likely-pathogenic *TPP1* variants confirm CLN2 diagnosis in patients aged 2 to 4 years presenting language delay/regression and epileptic seizures. Considering these recent local criteria, 20 out of 21 patients with 2 *TPP1* variants identified through the Epilepsy and Genetics Program could already have a confirmed CLN2 diagnosis. Patients hosting 2 different *TPP1* variants should be further evaluated to determine if the variants were found *in cis* or *in trans* ( *n* = 8 patients), while variants in homozygous patients could be inferred as biallelic ( *n* = 12 patients).

Biochemical analysis of TPP1 should be used to determine the impact of specific variants on the activity of the enzyme, in these cases of heterozygosity or presence of VUS, for example. As a diagnostic tool for CLN2, it may be useful mainly in cases in which the physician already has a strong suspicion of the disease. Some limitations should be considered:

- DBSs are practical samples that may be easily collected and transported to a reference laboratory for enzymatic analysis; however, deficient TPP1 results in DBS must be confirmed by molecular analysis for CLN2 diagnosis;
- Determination of TPP1 activity in leukocytes or fibroblasts are confirmatory tests, but demand a more structured laboratory to collect and prepare the samples, which cannot be easily sent to more distant reference laboratories, due to the need of specific transportation conditions and regulatory issues (if the reference laboratory is in a different country, which is common in Latin America).

In countries with a large territorial extension, such as Brazil, it may be more viable and effective to investigate genetic epileptic encephalopathies through a genetic panel that includes CLN and other genetic conditions, in a more comprehensive manner, than to perform TPP1 enzymatic analysis as the initial laboratory test. Usually, a history of unprovoked seizures and language delay, for example, do not lead to a specific suspicion of CLN2 disease, neither of NCL, for non-specialist pediatricians and pediatric neurologists. In these cases, it is recommended that the clinician should order an epilepsy gene panel to narrow down diagnostic possibilities. 15

Pathogenic or likely-pathogenic *TPP1* variants are a relatively common finding in NGS panels performed to investigate pediatric seizures, 21 22 23 emphasizing that these genetic panels can be a very useful tool to confirm or exclude CLN2 diagnosis and, if confirmed, provide disease-specific treatment for the patients.
