# Supplementary material for: A needle in a haystack? The impact of a targeted epilepsy gene panel in the identification of a treatable but rapidly progressive metabolic epilepsy: CLN2 disease
Source: Arq Neuropsiquiatr. 2024 May 19;82(5):s00441786854. doi: 10.1055/s-0044-1786854 (PMC11102811; doi:10.1055/s-0044-1786854)
Supplement: Supplementary file 1 — Supplementary Material [file 10-1055-s-0044-1786854-s240016.pdf]

Supplementary Material Chart 1 Molecular diagnostic panel with genes related to some epileptic syndromes

Symbol	Diseases
ADSL	Adenylosuccinase Deficiency
AFG3L2	Spastic Ataxia<br>Spinocerebellar Ataxia (SCA)
ALDH5A1	Succinic Semialdehyde Dehydrogenase Deficiency
ALDH7A1	Pyridoxine-Responsive Epilepsy
ALG13	Epileptic Encephalopathy
ARHGEF9	Early Infantile Epileptic Encephalopathy
ARSA	Metachromatic Leukodystrophy
ARX	Agenesis of Corpus Callosum and Abnormal Genitalia<br>Lissencephaly<br>Intellectual Deficiency and Epilepsy<br>Early Infantile Epileptic Encephalopathy<br>Partington X-Linked Intellectual Deficiency Syndrome
ATM	Ataxia-Telangiectasia<br>Breast Cancer
ATP1A2	Encephalopathy<br>Alternating Hemiplegia of Childhood<br>Hemiplegic Migraine
ATP1A3	Dystonia<br>Cerebellar Ataxia, Areflexia, Pes Cavus, Optic Atrophy, and Sensorineural Hearing Loss<br>Alternating Hemiplegia of Childhood
ATRX	Alpha-Thalassemia Myelodysplasia Syndrome<br>Intellectual Deficiency<br>Alpha-Thalassemia/Intellectual Deficiency Syndrome<br>Intellectual Deficiency-Hypotonic Facies Syndrome
BRAT1	Lethal Neonatal Rigidity and Multifocal Seizure Syndrome<br>Neurodevelopmental Disorder<br>Cerebellar Atrophy<br>Epilepsy
BSCL2	Congenital Generalized Lipodystrophy<br>Progressive Encephalopathy and Lipodystrophy<br>Spastic Paraplegia (SPG)<br>Distal Motor Neuronopathy
C12orf57	Temtamy Syndrome
CACNA1A	Episodic Ataxia<br>Hemiplegic Migraine<br>Epileptic Encephalopathy
CACNA2D2	Cerebellar Atrophy, Epilepsy, and Intellectual Deficiency
CARS2	Combined Oxidative Phosphorylation Deficiency
CASK	FG Syndrome<br>Intellectual Deficiency, Microcephaly, Pontocerebellar Hypoplasia
CDKL5	Intellectual Deficiency and/or Autism<br>Epileptic Encephalopathy
CHD2	Childhood-Onset Epileptic Encephalopathy<br>Intellectual Deficiency and/or Autism
CHRNA2	Nocturnal Frontal Lobe Epilepsy
CHRNA4	Nocturnal Frontal Lobe Epilepsy
CHRNB2	Nocturnal Frontal Lobe Epilepsy
CLCN4	Intellectual Deficiency and Epilepsy
CLN3	Ceroid Lipofuscinosis (CLN)
CLN5	Ceroid Lipofuscinosis (CLN)

(*Continued*)

 © 2024. The Author(s).

Supplementary Material Chart 1 (*Continued*)

Symbol	Diseases
CLN6	Ceroid Lipofuscinosis (CLN)<br>Ceroid Lipofuscinosis (CLN)
CLN8	Ceroid Lipofuscinosis (CLN)<br>Ceroid Lipofuscinosis (CLN)
CNTNAP2	Cortical Dysplasia-Focal Epilepsy Syndrome
CSTB	Myoclonic Epilepsy of Unverricht and Lundborg
CTSD	Ceroid Lipofuscinosis (CLN)<br>Microcephaly
DCX	Lissencephaly
DEPDC5	Focal Epilepsy with Variable Foci
DNAJC5	Ceroid Lipofuscinosis (CLN)
DNM1	Epileptic Encephalopathy<br>Intellectual Deficiency and/or Autism
DOCK7	Epileptic Encephalopathy
DYRK1A	Intellectual Deficiency
EEF1A2	Intellectual Deficiency<br>Epileptic Encephalopathy
EFHC1	Myoclonic Juvenile Epilepsy<br>Juvenile Absence Epilepsy
EHMT1	Kleefstra Syndrome
EPM2A	Myoclonic Epilepsy of Lafora
FA2H	Spastic Paraplegia (SPG)
FARS2	Combined Oxidative Phosphorylation Deficiency
FOLR1	Neurodegeneration Due To Cerebral Folate Transport Deficiency
FOXG1	Microcephaly<br>Congenital Variant Rett Syndrome
FRRS1L	Epileptic Encephalopathy
GABBR2	Intellectual Deficiency<br>Epileptic Encephalopathy<br>Neurodevelopmental Disorder
GABRA1	Idiopathic Generalized Epilepsy<br>Epileptic Encephalopathy
GABRB2	Epileptic Encephalopathy<br>Intellectual Deficiency and/or Autism
GABRB3	Childhood Absence Epilepsy<br>Intellectual Deficiency and/or Autism
GABRG2	Childhood Absence Epilepsy<br>Generalized Epilepsy and Febrile Seizures Plus<br>Epileptic Encephalopathy
GAMT	Cerebral Creatine Deficiency
GATM	Cerebral Creatine Deficiency
GLB1	GM1-Gangliosidosis<br>Mucopolysaccharidosis
GLRA1	Hyperekplexia
GNAO1	Neurodevelopmental Disorder with Involuntary Movements<br>Epileptic Encephalopathy
GOSR2	Progressive Myoclonic Epilepsy

 © 2024. The Author(s).

Supplementary Material Chart 1 (*Continued*)

Symbol	Diseases
GRIN1	Neurodevelopmental Disorder, Hyperkinetic Movements, and Epilepsy<br>Polymicrogyria<br>Intellectual Deficiency
GRIN2A	Intellectual Deficiency and Epileptic Encephalopathy<br>Focal Epilepsy, Speech Disorder, and Mild Intellectual Deficiency
GRIN2B	Intellectual Deficiency<br>Epileptic Encephalopathy
GRN	Ceroid Lipofuscinosis (CLN)<br>Frontotemporal Dementia (FTD)
HCN1	Epileptic Encephalopathy<br>Generalized Epilepsy with Febrile Seizures Plus
HNRNPU	Epileptic Encephalopathy<br>Intellectual Deficiency and/or Autism
IER3IP1	Microcephaly, Epilepsy, and Diabetes Syndrome
IQSEC2	Intellectual Deficiency
ITPA	Inosine Triphosphatase Deficiency<br>Epileptic Encephalopathy<br>Martsolf Syndrome
JMJD1C	Rett syndrome*<br>Intellectual disability*
KANSL1	Koolen-De Vries Syndrome
KCNA2	Epileptic Encephalopathy
KCNB1	Epileptic Encephalopathy
KCNC1	Progressive Myoclonic Epilepsy<br>Intellectual Deficiency and/or Autism
KCNH2	Short-QT Syndrome<br>Long-QT Syndrome
KCNJ10	Epilepsy, Sensorineural Deafness, Ataxia, Intellectual Deficiency, and Electrolyte Imbalance
KCNMA1	Developmental Delay, Cerebellar Hypoplasia, and Myoclonic Epilepsy<br>Generalized Epilepsy and Paroxysmal Dyskinesia
KCNQ2	Benign Familial Neonatal Epilepsy<br>Epileptic Encephalopathy<br>Intellectual Deficiency and/or Autism
KCNQ3	Intellectual Deficiency and/or Autism<br>Benign Familial Neonatal Epilepsy
KCNT1	Epileptic Encephalopathy<br>Nocturnal Frontal Lobe Epilepsy
KCTD7	Progressive Myoclonic Epilepsy and Intracellular Inclusions
LGI1	Temporal Lobe Epilepsy
LIAS	Hyperglycinemia, Lactic Acidosis, and Epilepsy
LMNB2	Acquired Partial Lipodystrophy<br>Progressive Myoclonic Epilepsy
MBD5	Intellectual Deficiency
MECP2	Neonatal Severe Encephalopathy<br>Rett Syndrome
MEF2C	Intellectual Deficiency
MFSD8	Ceroid Lipofuscinosis (CLN)<br>Macular Dystrophy and Central Cone Involvement
MTOR	Smith-Kingsmore Syndrome
NEDD4L	Periventricular Nodular Heterotopia

(*Continued*)

 © 2024. The Author(s).

Supplementary Material Chart 1 (*Continued*)

Symbol	Diseases
NEXMIF	Intellectual Deficiency
NGLY1	Congenital Disorder of Deglycosylation
NHLRC1	Myoclonic Epilepsy of Lafora
NPRL3	Focal Epilepsy with Variable Foci
NRXN1	Pitt-Hopkins-Like Syndrome
PACS1	Schuurs-Hoeijmakers syndrome
PCDH19	Epileptic Encephalopathy
PIGA	Multiple Congenital Anomalies-Hypotonia-Epilepsy Syndrome
PIGN	Multiple Congenital Anomalies-Hypotonia-Epilepsy Syndrome
PIGO	Hyperphosphatasia and Intellectual Deficiency Syndrome
PLA2G6	Neurodegeneration and Brain Iron Accumulation (NBIA)<br>Parkinson's Disease
PLCB1	Epileptic Encephalopathy
PNKD	Paroxysmal Nonkinesigenic Dyskinesia
PNKP	Microcephaly, Epilepsy, and Developmental Delay<br>Ataxia-Oculomotor Apraxia
PNPO	Pyridoxamine 5-Prime-Phosphate Oxidase Deficiency
POLG	Mitochondrial DNA Depletion Syndrome<br>Progressive External Ophthalmoplegia and Mitochondrial DNA Deletions<br>Sensory Ataxic Neuropathy, Dysarthria, and Ophthalmoparesis<br>Mitochondrial DNA Depletion Syndrome<br>Progressive External Ophthalmoplegia and Mitochondrial DNA Deletions
PPT1	Ceroid Lipofuscinosis (CLN)
PRDM8	Progressive Myoclonic Epilepsy
PRICKLE1	Progressive Myoclonic Epilepsy
PRIMA1	Sleep-related Hypermotor Epilepsy*
PRRT2	Episodic Kinesigenic Dyskinesia<br>Infantile Convulsions and Paroxysmal Choreoathetosis<br>Benign Familial Infantile Epilepsy
PSAP	Metachromatic Leukodystrophy<br>Atypical Gaucher Disease<br>Combined Saposin Deficiency<br>Atypical Krabbe Disease
PURA	Intellectual Deficiency
QARS1	Progressive Microcephaly, Epilepsy, and Cerebral-Cerebellar Atrophy
RELN	Lissencephaly
ROGDI	Kohlschutter-Tonz Syndrome
RPIA	Ribose-5-Phosphate Isomerase Deficiency
SATB2	Glass Syndrome
SCARB2	Progressive Myoclonic Epilepsy and Renal Failure
SCN1A	Generalized Epilepsy and Febrile Seizures Plus<br>Epileptic Encephalopathy<br>Hemiplegic Migraine
SCN1B	Epileptic Encephalopathy<br>Generalized Epilepsy and Febrile Seizures Plus<br>Brugada Syndrome<br>Atrial Fibrillation

 © 2024. The Author(s).

Supplementary Material Chart 1 (*Continued*)

Symbol	Diseases
SCN2A	Epileptic Encephalopathy<br>Benign Familial Infantile Epilepsy<br>Intellectual Deficiency and/or Autism
SCN3A	Epileptic Encephalopathy<br>Focal Epilepsy with Variable Foci
SCN8A	Epileptic Encephalopathy<br>Impairment and Cerebellar Ataxia<br>Non-Progressive Myoclonus
SCN9A	Congenital Indifference To Pain<br>Primary Erythermalgia<br>Paroxysmal Extreme Pain Disorder<br>Generalized Epilepsy and Febrile Seizures Plus
SERPINI1	Encephalopathy and Neuroserpin Inclusion Bodies
SGCE	Myoclonic Dystonia
SIK1	Epileptic Encephalopathy
SLC12A5	Epileptic Encephalopathy<br>Idiopathic Generalized Epilepsy
SLC13A5	Epileptic Encephalopathy
SLC19A3	Thiamine Metabolism Dysfunction Syndrome
SLC25A12	Global Cerebral Hypomyelination
SLC25A22	Epileptic Encephalopathy
SLC2A1	GLUT1 Deficiency Syndrome<br>Dystonia<br>Stomatin-Deficient Cryohydrocytosis and Neurologic Defects<br>Idiopathic Generalized Epilepsy
SLC35A2	Congenital Disorder of Glycosylation (CDG)
SLC6A1	Myoclonic-Atonic Epilepsy<br>Intellectual Deficiency and/or Autism
SLC6A8	Cerebral Creatine Deficiency
SLC9A6	Syndromic Intellectual Deficiency
SMC1A	Cornelia De Lange Syndrome
SNX27	Epileptic Encephalopathy, Early Infantile, 6 and Epilepsy*
SPATA5	Epilepsy, Hearing Loss, and Intellectual Deficiency
SPTAN1	Epileptic Encephalopathy
ST3GAL5	Amish Infantile Epilepsy Syndrome
STRADA	Polyhydramnios, Megalencephaly, and Epilepsy
STX1B	Generalized Epilepsy and Febrile Seizures Plus
STXBP1	Epileptic Encephalopathy<br>Intellectual Deficiency and/or Autism
SYN1	Epilepsy, Intellectual Deficiency, and Behavior Disorders
SYNGAP1	Intellectual Deficiency
SYNJ1	Early-Onset Parkinson's Disease<br>Epileptic Encephalopathy
SZT2	Epileptic Encephalopathy
TBC1D24	Deafness, Onychodystrophy, Osteodystrophy, Intellectual Deficiency, and Epilepsy<br>Epileptic Encephalopathy & Infantile Myoclonic Epilepsy
TCF4	Pitt-Hopkins Syndrome

(*Continued*)

 © 2024. The Author(s).

Supplementary Material Chart 1 (*Continued*)

Symbol	Diseases
TPK1	Thiamine Metabolism Dysfunction Syndrome
TPP1	Ceroid Lipofuscinosis (CLN)<br>Spinocerebellar Ataxia (SCA)
TSC1	Tuberous Sclerosis<br>Lymphangioleiomyomatosis<br>Focal Cortical Dysplasia of Taylor
TSC2	Lymphangioleiomyomatosis<br>Tuberous Sclerosis
TWNK	Mitochondrial DNA Depletion Syndrome<br>Perrault Syndrome<br>Progressive External Ophthalmoplegia and Mitochondrial DNA Deletions
UBE3A	Angelman Syndrome
WDR45	Neurodegeneration and Brain Iron Accumulation (NBIA)
WWOX	Spinocerebellar Ataxia (SCAR)<br>Epileptic Encephalopathy<br>Esophageal Cancer
ZDHHC9	Syndromic Intellectual Deficiency
ZEB2	Mowat-Wilson Syndrome

*These diseases are not clearly associated with this gene in OMIM.

 © 2024. The Author(s).
